# Multidimensional characterization of vegetable breads using multisource intelligent sensory technologies and feature-level data fusion

**DOI:** 10.3389/fnut.2026.1970901

**Published:** 2026-10-13

**Authors:** Yongfeng Jiang, Han Yang, Cuiying Jian, Yaoxu Lin, Enrui Hu, Hao Zhang, Qingjie Shi, Yuwen Yi

**Affiliations:** 1 School of Food Engineering, Guangdong Teachers College of Foreign Languages and Arts, Guangzhou, China; 2 College of Culinary and Food Science Engineering, Sichuan Tourism University, Chengdu, China; 3 Cuisine Science Key Laboratory of Sichuan Province, Sichuan Tourism University, Chengdu, China; 4 Sichuan Digital Economy Industry Development Research Institute, Chengdu, China

**Keywords:** electronic eye, electronic nose, electronic tongue, feature-level data fusion, multisource intelligent sensing, vegetable breads

## Abstract

**Background:**

Food sensory quality is multidimensional, and a single intelligent sensory technique cannot simultaneously characterize color, taste, and odor across multiple dimensions. Multisource intelligent sensory analysis combined with data fusion provides an approach for the objective characterization of multidimensional food sensory characteristics.

**Methods:**

Control bread (CK) and four vegetable breads prepared with pumpkin (PU), *Pueraria thomsonii* (PG), red pepper (RP), and celery (CE) were analyzed using an electronic eye, an electronic tongue, and an ultrafast gas chromatography electronic nose. These instruments provided color, taste-response, and volatile-compound information, respectively. Principal component analysis (PCA), differential-peak screening, and tentative assignment based on dual-column retention-index (RI) matching were used to compare characteristics among formulations. Principal components from each single-modality dataset were then retained for feature-level data fusion followed by PCA.

**Results:**

The vegetable juices produced distinct color, taste-response, and volatile-compound characteristics in the breads. The first three electronic-eye principal components explained 91.95% of the variance. PU was dominated by strong yellow and vivid yellow, RP showed a clear orange-yellow appearance, and CE retained a green hue. The first two electronic-tongue principal components explained 90.03% of the variance. PG showed relatively high umami and bitterness response indices, whereas CE showed a relatively high saltiness response index. The first five principal components of the electronic-nose data explained 82.89% of the variance, and 17 volatile compounds were tentatively assigned. PG showed high responses for several fermentation-related volatiles. A peak tentatively assigned to acetone was detected only in RP under the current analytical conditions, whereas peaks tentatively assigned to myrcene and β-phellandrene showed pronounced responses in CE. After feature-level data fusion, the first three principal components explained 80.78% of the variance. Loading analysis showed that multiple latent variables from the electronic eye, electronic tongue, and electronic nose jointly contributed to the multidimensional characterization of the formulations.

**Conclusion:**

Breads prepared with different vegetable juices exhibited distinct characteristics in terms of color, taste-response profiles, and volatile-compound profiles. Feature-level fusion of multisource intelligent sensory data enables the joint representation of information from these three instrumental dimensions within a unified analytical framework, providing a methodological framework for the multidimensional characterization of vegetable breads.

## 1 Introduction

Food sensory characteristics are inherently multidimensional. Color, odor, and taste jointly shape human perception and judgment of foods. Conventional sensory evaluation directly reflects human sensory experience and remains essential for assessing perceived food quality. However, its results may also be influenced by assessors’ experience, physiological status, and environmental conditions. These factors may reduce the stability and repeatability of sensory evaluation, particularly when many samples are evaluated or when differences among samples are subtle. Instrumental sensory techniques can therefore provide complementary objective information for characterizing different dimensions of food quality. With advances in sensor technology and chemometrics, electronic eyes, electronic tongues, and electronic noses have been increasingly used in food quality research. These techniques provide instrument-based information related to color, taste, and odor, respectively, and offer objective tools for examining different sensory dimensions of foods (1–3).

However, color, taste, and odor have different physicochemical bases. A single intelligent sensory technique generally captures only one dimension of sample variation and therefore cannot fully describe differences across multiple sensory attributes. The electronic eye primarily characterizes color composition and distribution, the electronic tongue reflects taste-response characteristics, and the electronic nose characterizes volatile-compound profiles. Because these technologies differ in their information sources and response mechanisms, they can provide complementary information from different dimensions (2–4). Data fusion can further integrate features acquired from different platforms within a common analytical framework and improve the joint analysis of multidimensional food characteristics (3, 5–7). Electronic eyes, electronic tongues, and electronic noses have already been used in combination for quality identification and characterization of tea, medicinal materials, and other foods (5–8). These studies demonstrate the potential of multisource intelligent sensory technologies and data fusion for comprehensive food characterization.

Bread provides a relatively stable food matrix and a readily controlled processing system, making it suitable for comparing the effects of different vegetable ingredients. When the basic formulation and processing conditions are kept constant, using different vegetable juices as the liquid component allows the effects of the vegetable ingredients to be compared within a common bread-processing framework. Previous studies have shown that vegetable juices or other vegetable-derived ingredients can alter bread color, physicochemical properties, and sensory characteristics (9–11). After vegetable ingredients enter the same bread matrix, the compounds they introduce are further affected by the dough matrix, fermentation, and baking. Consequently, the final color, taste-response, and volatile-compound characteristics cannot be inferred directly from those of the raw vegetables. Incorporating different vegetable juices into a common bread-processing framework therefore allows their resulting multidimensional characteristics to be compared under consistent formulation and processing conditions, although the chemical composition of the individual vegetable juices was not standardized.

Accordingly, control bread and breads prepared with pumpkin, *Pueraria thomsonii*, red pepper, and celery juices were investigated to determine how breads prepared with different vegetable juices exhibited multidimensional instrumental characteristics after undergoing the same bread-making process. An electronic eye, electronic tongue, and ultrafast gas chromatography electronic nose were used to characterize color composition, taste-response profiles, and volatile-compound characteristics, respectively. Within a common bread-processing framework, this study examined whether, after the breads underwent the same fermentation and baking process, differences among the vegetable-juice formulations were expressed consistently across three instrumental dimensions—color composition, taste-response profiles, and volatile-compound profiles—or whether they exhibited dimension-specific patterns. The scope of the present study was limited to multidimensional instrumental characterization rather than consumer acceptance or sensory preference, and no direct equivalence between instrumental responses and human sensory perception was assumed. Feature-level data fusion was then used to integrate these dimension-specific patterns within a common data space for joint multidimensional characterization. The findings provide insights into the formation of multidimensional instrumental differences associated with different vegetable-juice formulations and offer a methodological framework for objective characterization of vegetable-based bakery products.

## 2 Materials and methods

### 2.1 Materials and instruments

#### 2.1.1 Materials and reagents

High-gluten wheat flour, Angel high-sugar active dry yeast, granulated sugar, table salt, butter, milk powder, and eggs were purchased commercially. Pumpkin, *Pueraria thomsonii*, red pepper, and celery were purchased from Jinyan Farmers’ Market. Quinine hydrochloride, sucrose, and tannic acid were used for electronic-tongue evaluation of bitterness, sweetness, and astringency, respectively. An n-alkane (C5-C16) standard solution was used to calibrate retention indices for the electronic nose.

#### 2.1.2 Major instruments and equipment

The main analytical instruments were a Heracles NEO 300G ultrafast gas chromatography electronic nose, an Astree LS48 electronic tongue, and an IRIS VA400 electronic eye (Alpha MOS, France). Bread production was carried out using a GC-1065P11567 oven with a proofing chamber (Huizhou Gaobi Baking Equipment Co., Ltd., China) and an RN20/VL 2 mixer (20 L; Shenzhen Siruida Food Equipment Co., Ltd., China). Other standard laboratory equipment was also used.

### 2.2 Experimental methods

#### 2.2.1 Preparation of vegetable breads

Vegetable breads were prepared according to the vegetable-juice bread method described by Raczyk et al. (9), with modifications to the formulation and processing conditions used in the present study. All ingredient percentages were calculated based on flour weight (100%). The basic formulation consisted of 1.5% dry yeast, 15% granulated sugar, 1.5% salt, 6% butter, 4% milk powder, 8% egg, and 60% liquid (vegetable juice for vegetable breads or water for CK). Pumpkin, *Pueraria thomsonii*, red pepper, and celery were washed, cut into pieces, homogenized with water at a raw material-to-water mass ratio of 1:1 (w/w), meaning that 100 g of vegetable material was homogenized with 100 g of water, and filtered to obtain the corresponding juices.

High-gluten wheat flour, dry yeast, milk powder, granulated sugar, egg, and the corresponding liquid (vegetable juice for vegetable breads or water for CK) were mixed for 2 min at low speed and 6 min at high speed. Salt and butter were then added, followed by mixing for 2 min at low speed and 3 min at high speed. The dough underwent bulk fermentation at 35 °C and 75% relative humidity for 60 min, at which point its volume was approximately twice the initial volume. The fermented dough was divided into 60-g portions, rounded, degassed, and allowed to rest for 20 min. After shaping, the dough was proofed at 35 °C and 75% relative humidity for 45 min. The breads were then baked for 18 min with upper and lower oven temperatures of 210 and 170 °C, respectively. Immediately after baking, the loaves were removed from the oven and cooled naturally on a rack at room temperature (20–22 °C) for 60 min, after which the samples were immediately prepared for the three instrumental analyses without additional storage.

Five formulations were prepared: control bread (CK), pumpkin bread (PU), *Pueraria thomsonii* bread (PG), red pepper bread (RP), and celery bread (CE). In each vegetable bread formulation, the corresponding vegetable juice served as the liquid component at 60% of the flour weight. CK received an equal amount of water instead of vegetable juice, while all other formulation and processing conditions were kept constant. Three independent production batches were prepared for each formulation. Samples from the same production batch were used for all three instrumental analyses, and the datasets were matched by production batch for subsequent feature-level data fusion.

#### 2.2.2 Electronic eye analysis

An IRIS VA400 electronic eye (Alpha MOS, France) was operated with a 5-mm aperture and D65 illumination. Bread from each independent production batch was cut through the center, and the cross section was placed face up on a blue background. Images were acquired under both top and bottom illumination. AlphaSoft software was used to remove the blue background, define the complete bread cross section as the color-analysis region, identify the color-code composition, and calculate the relative area proportion of each color code (12). One cross-sectional image was acquired from each independent production batch, yielding three images per formulation for color analysis.

#### 2.2.3 Electronic tongue analysis

Bread from each independent production batch was ground for 10 s. A 1.0-g portion was mixed with 50 mL of purified water and sonicated for 10 min. The suspension was centrifuged in a TD5A-WS centrifuge (Xiangyi, China; 12 × 15-mL angle rotor; maximum rotor radius, 125 mm) at 5,000 rpm (approximately 3,494 × g) for 15 min and then filtered. A 25-mL aliquot of the filtrate was used for measurement. An Astree LS48 electronic tongue (Alpha MOS, France) equipped with AHS, PKS, CTS, NMS, CPS, ANS, and SCS sensors and an Ag/AgCl reference electrode was used. Before analysis, the sensors underwent activation, calibration, and diagnostic procedures using hydrochloric acid (0.01 mol/L), sodium chloride, and monosodium glutamate solutions. The sensor responses recorded at the end of the 120-s acquisition period were used for data analysis. Each independent production batch was measured seven consecutive times. When the relative standard deviation (RSD) of the seven measurements was <5%, the mean of the seven measurements was used as the batch value, giving three independent production-batch values for each formulation. After each measurement, the sensors were rinsed with deionized water for 120 s. Samples were randomly assigned to even-numbered positions in the autosampler, while adjacent odd-numbered positions were filled with deionized water to minimize carryover and potential sequence effects during consecutive measurements.

The Taste Ranking function built into the electronic tongue software was used to evaluate sourness, saltiness, umami, bitterness, sweetness, and astringency. Standard-addition mode was used for bitterness, sweetness, and astringency. Quinine hydrochloride at 0.01, 0.05, and 0.10% was assigned values of 1, 5, and 10, respectively. Sucrose at 0.125, 1.25, and 2.5% was assigned values of 1, 10, and 20, respectively. Tannic acid at 0.01, 0.05, and 0.10% was assigned values of 1, 5, and 10, respectively. Each taste-response index was used only to compare formulations within the same taste attribute.

#### 2.2.4 Ultrafast gas chromatography electronic nose analysis

Ground bread (1.00 g) was placed in a 20-mL headspace vial, sealed with a PTFE septum, and equilibrated with agitation at 60 °C for 20 min. Samples were then analyzed using a Heracles NEO 300G ultrafast gas chromatography electronic nose (Alpha MOS, France). The headspace injection volume was 5,000 μL, the injection rate was 125 μL/s, the injector temperature was 200 °C, and the injection duration was 45 s. The trap temperature was programmed from an initial 40 °C to a final 240 °C. The split flow was 10 mL/min, and the trapping time was 50 s. The MXT-5 (low-polarity) and MXT-1701 (medium-polarity) columns were operated simultaneously. The column temperature was increased from 50 to 120 °C at 0.5 °C/s and then to 250 °C at 3.0 °C/s. The acquisition time was 253 s, the FID temperature was 260 °C, and the detector gain was 12. One headspace sample was prepared and injected once for each independent production batch, yielding three independent production-batch measurements per formulation.

AlphaSoft software was used for peak detection, integration, and alignment. The minimum peak height was 40, the minimum peak area was 50, the signal-to-noise ratio was 3, the peak width was 1 s, and the RI tolerance for peak alignment was 10 RI units. Retention indices were calibrated with C_5_-C_16_ n-alkanes analyzed under the same conditions. Volatile compounds were tentatively assigned by matching experimental RIs on the MXT-5 and MXT-1701 columns with reference RIs in the AroChemBase database. The allowable deviation was ±20 RI units for each column. When multiple candidate compounds were available for the same chromatographic peak, the candidate with the smallest cumulative RI difference across the two columns was given priority. Because neither mass spectrometry nor authentic standards were used for structural confirmation, all database-based assignments were treated as tentative identifications.

### 2.3 Data processing and statistical analysis

For electronic-eye data, low-abundance color codes with a mean relative area proportion <1% in all five formulations were removed, and the retained variables were autoscaled before PCA. For each formulation, color codes with a mean relative area proportion ≥5% across the three independent production batches were defined as major characteristic colors and used for color composition analysis. For the electronic tongue, the endpoint responses of the seven sensors were autoscaled before heatmap generation, PCA, and subsequent feature-level data fusion. The six taste-response indices generated by Taste Ranking were used only for descriptive comparisons among formulations within the same taste attribute and were not included in PCA or data fusion. For the electronic-tongue sensor responses, effect sizes were additionally quantified using eta squared (η^2^), calculated as the ratio of the between-group sum of squares to the total sum of squares.

The electronic-nose dataset initially contained 123 aligned chromatographic peaks. Twenty-six variables showing no variation across the 15 independent production batches were removed, leaving 97 effective peaks. This variable exclusion was based solely on variation across the complete dataset and did not use formulation labels. Peak areas were transformed using ln(1 + x), autoscaled, and subjected to unsupervised PCA to characterize overall differences in volatile-compound profiles. The resulting PCA scores served as the electronic-nose input for subsequent feature-level data fusion. Fisher scores were additionally used to rank differential peaks, and the top 20 chromatographic peaks were selected for differential-variable interpretation and tentative identification by dual-column RI matching. Because formulation labels were used to calculate Fisher scores, PCA based on the top 20 peaks was used only to explore associations between differential variables and sample distributions. It was not considered independent evidence of sample-discrimination performance.

For feature-level data fusion, PCA was performed separately on the electronic-eye, electronic-tongue, and full-peak electronic-nose datasets. A cumulative explained-variance threshold of ≥80% was specified in advance, and the minimum number of principal components required to reach this threshold was retained from each modality. The retained principal-component scores from each modality were arranged as a score matrix *T_m_*, where the rows represented the 15 matched production batches and the columns represented the retained principal components of modality *m*. To reduce the influence of differences in block dimensionality and total variance among modalities, block-wise Frobenius normalization was applied to each modality-specific score matrix as a whole (13), as shown in Equation 1:

$${\tilde{T}}_{m}=\frac{T_{m}}{{\left\|T_{m}\right\|}_{F}}$$ (1)

where ${\tilde{T}}_{m}\;$ is the normalized score matrix for modality m, T_m_ is the original PCA score matrix, and ||T_m_ ||_F_ is the Frobenius norm of the entire score matrix, calculated as shown in Equation 2:

$${\left\|T_{m}\right\|}_{F}=\sqrt{\sum_{i=1}^{n}\sum_{j=1}^{k_{m}}t_{\mathrm{ij}}^{2}}$$ (2)

where t_ij_ denotes the score of production batch *i* on principal component *j*, *n* = 15 is the total number of matched production batches, and *k_m_* is the number of retained principal components for modality *m*. Thus, all elements within a given modality-specific score matrix were divided by the same Frobenius norm; Frobenius normalization was not performed separately for individual PC columns. The three normalized score matrices were then concatenated side-by-side after aligning their rows according to the matched production batches. PCA was performed directly on the concatenated matrix without additional columnwise standardization, thereby preserving the relative variance structure of the latent variables after block normalization (13).

The independent production batch was used as the experimental unit, with *n* = 3 for each formulation. For univariate outcomes with independent batch-level data and inferential testing, results were reported as mean ± SD. Each variable was first analyzed by one-way analysis of variance (ANOVA) to test the overall formulation effect. When multiple variables were tested simultaneously within the same analytical module, *p* values from the corresponding ANOVAs were adjusted using the Benjamini-Hochberg procedure to control the false discovery rate (FDR). An FDR-adjusted *p* < 0.05 was used to define a significant overall formulation effect. Variables with a significant overall formulation effect were further compared among formulations using Tukey’s multiple-comparison test, with *p* < 0.05 considered significant. For single tests that did not require correction for multiple variables, *p* < 0.05 was used as the significance threshold. For candidate electronic-nose compounds with nondetects and incomplete data across the 15 independent production batches, nondetected values were not imputed as zero. ANOVA and FDR adjustment were not performed for these compounds, which were compared descriptively only. Because each formulation included only three independent production batches, PCA and feature-level data fusion results were interpreted as exploratory multivariate analyses. Data organization and preliminary processing were conducted using AlphaSoft and Excel. Statistical analyses and figures were prepared using SPSS 25.0 and Origin 2026.

## 3 Results and discussion

### 3.1 Electronic eye analysis

As shown in Figure 1, CK was off-white with only a slight pale-yellow hue, whereas PU was clearly yellow and relatively bright. PG was darker than CK and appeared grayish pale yellow. RP was orange-yellow, and CE was yellow-green. Overall, PU, RP, and CE showed more pronounced color characteristics. CK and PG had relatively similar hues but differed in lightness and grayness. Visual inspection primarily reflects macroscopic color differences in bread cross sections; therefore, the electronic eye was used to quantify the composition and relative area proportions of more detailed color codes.

**Figure 1:**
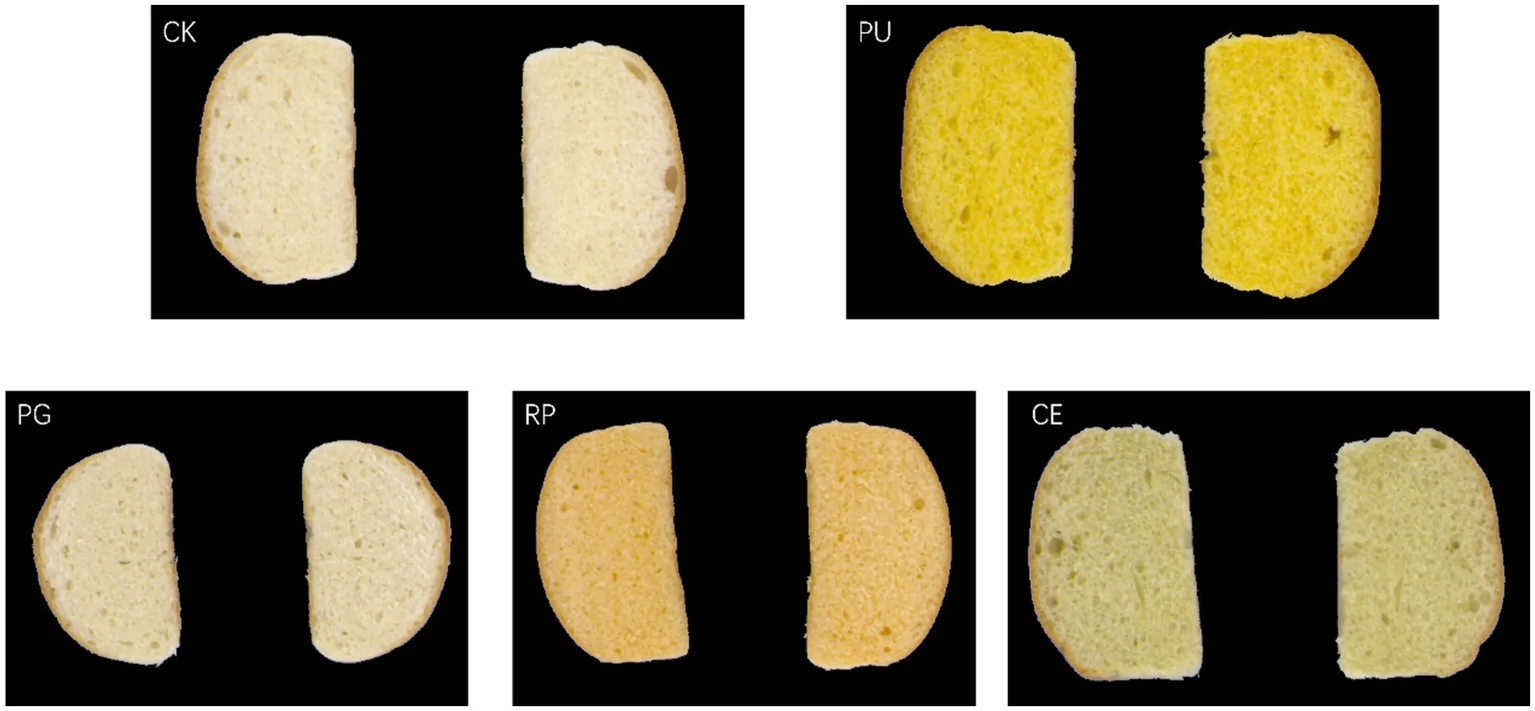
Electronic-eye images of vegetable breads. CK, control bread; PU, pumpkin bread; PG, *Pueraria thomsonii* bread; RP, red pepper bread; CE, celery bread.

The stacked plot of the relative area proportions of the major color codes is shown in Figure 2, and the corresponding statistical comparisons are provided in Supplementary Table S1. All 24 color codes showed significant formulation effects (FDR-adjusted *p* < 0.05). CK, PU, PG, RP, and CE contained 3, 7, 5, 6, and 5 major characteristic colors, respectively, accounting for 58.06, 54.36, 59.93, 53.34, and 53.82% of the corresponding cross-sectional areas. The major color codes of CK were concentrated in the pale-yellow range, with No. 3803 and No. 3802 together accounting for 52.73%. PU consisted of several strong yellow and vivid yellow color codes. Strong yellow accounted for 43.48% and vivid yellow for 10.88%, indicating that the yellow appearance of PU resulted from multiple yellow color codes rather than a single dominant code. PG shared the pale-yellow codes No. 3803 and No. 3802 with CK, but their combined proportion decreased to 29.08%. In contrast, the grayish yellow codes No. 3530 and No. 3529 together accounted for 24.98%. Their L* values were 79.36 and 79.03, respectively, lower than those of No. 3803 and No. 3802 in CK (85.61 and 85.28). This result was consistent with the darker appearance of PG observed visually.

**Figure 2:**
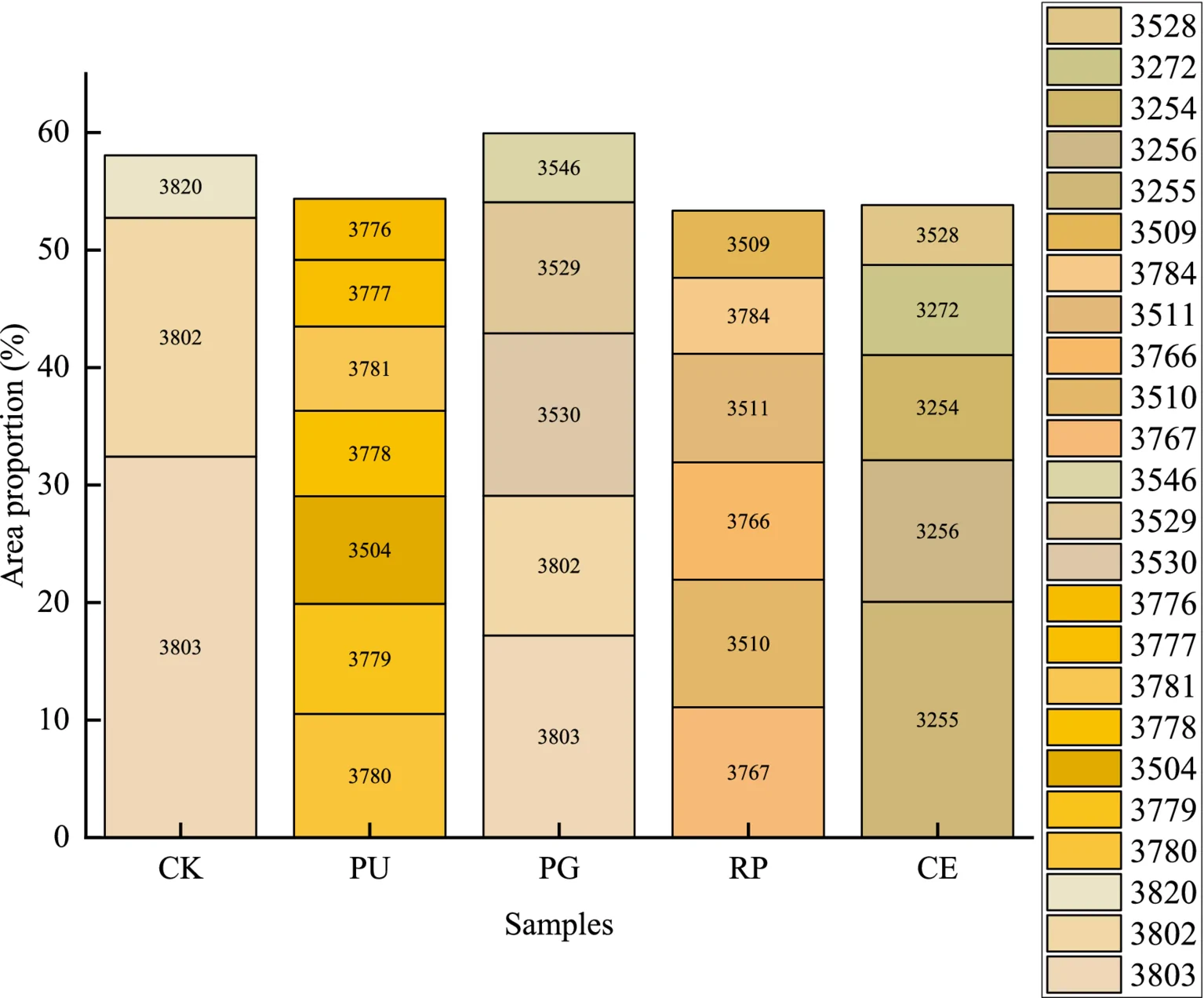
Relative area proportions of major characteristic colors in different vegetable breads. 3803, pale yellow; 3802, pale yellow; 3820, pale yellow green; 3780, strong yellow; 3779, strong yellow; 3504, strong yellow; 3778, strong yellow; 3781, strong yellow; 3777, vivid yellow; 3776, vivid yellow; 3530, grayish yellow; 3529, grayish yellow; 3546, pale greenish yellow; 3767, moderate orange yellow; 3510, moderate yellow; 3766, moderate orange yellow; 3511, moderate yellow; 3784, moderate yellow; 3509, strong yellow; 3255, moderate yellow; 3256, grayish yellow; 3254, moderate yellow; 3272, grayish greenish yellow; 3528, moderate yellow. For each formulation, only color codes with a mean relative area proportion ≥5% across the three independent production batches are shown. Bar height represents the sum of the area proportions of the displayed color codes and therefore does not equal 100%. CK, control bread; PU, pumpkin bread; PG, *Pueraria thomsonii* bread; RP, red pepper bread; CE, celery bread.

RP was composed mainly of moderate orange yellow, moderate yellow, and strong yellow. The moderate orange yellow codes No. 3767 and No. 3766 together accounted for 21.08%, and their a* values were 11.05 and 9.73, respectively, indicating a pronounced red component within the orange-yellow color. CE was dominated by moderate yellow (34.10%) and also contained 12.06% grayish yellow and 7.66% grayish greenish yellow. The a* value of No. 3272 was −10.49, indicating that CE retained a green hue despite its predominantly yellow appearance. Overall, the vegetable ingredients altered both the dominant crumb hue and the number and relative proportions of the major color categories.

These color differences may be related to the natural pigments introduced by the vegetable ingredients and their stability during fermentation and baking. Pumpkin is rich in carotenoids. Different carotenoid structures vary in thermal stability and can undergo different degrees of degradation during baking (14, 15), which may help explain why PU remained dominated by strong yellow and vivid yellow after baking. The principal pigments in red pepper include carotenoids such as capsanthin and capsorubin. Heating can cause carotenoid degradation, isomerization, and oxidative transformation, and red pigments may be lost to a greater extent than some yellow carotenoids (16). These changes may partly explain the shift of RP from the red color of the raw pepper toward orange-yellow after baking. The green coloration of celery is closely associated with chlorophyll-related pigments (17). Chlorophyll can lose magnesium and undergo degradation during heating, accompanied by a decrease in greenness (18). Thus, the predominantly yellow appearance of CE with some residual green hue may reflect both chlorophyll retention and thermal transformation. In contrast, PG was characterized mainly by lower lightness and a greater proportion of grayish yellow. Carotenoids, chlorophylls, and their thermal-transformation products were not directly measured in the breads. Therefore, these interpretations are based on the literature and represent possible sources of the observed color differences rather than direct evidence of pigment changes.

PCA of the selected color codes is shown in Figure 3. PC1, PC2, and PC3 explained 38.45, 29.84, and 23.66% of the variance, respectively, for a cumulative explained variance of 91.95%. The first three principal components were therefore retained for subsequent feature-level data fusion. The PCA included all 15 independent production batches from the five formulations. Color data from the three independent batches within each formulation were highly consistent, and their score points almost completely overlapped in the figure, indicating good batch-to-batch color consistency. PU, RP, and CE occupied different regions of the PCA space, whereas CK and PG were relatively close in color but remained distinguishable. These results show that the electronic eye effectively characterized crumb-color differences among the vegetable breads.

**Figure 3:**
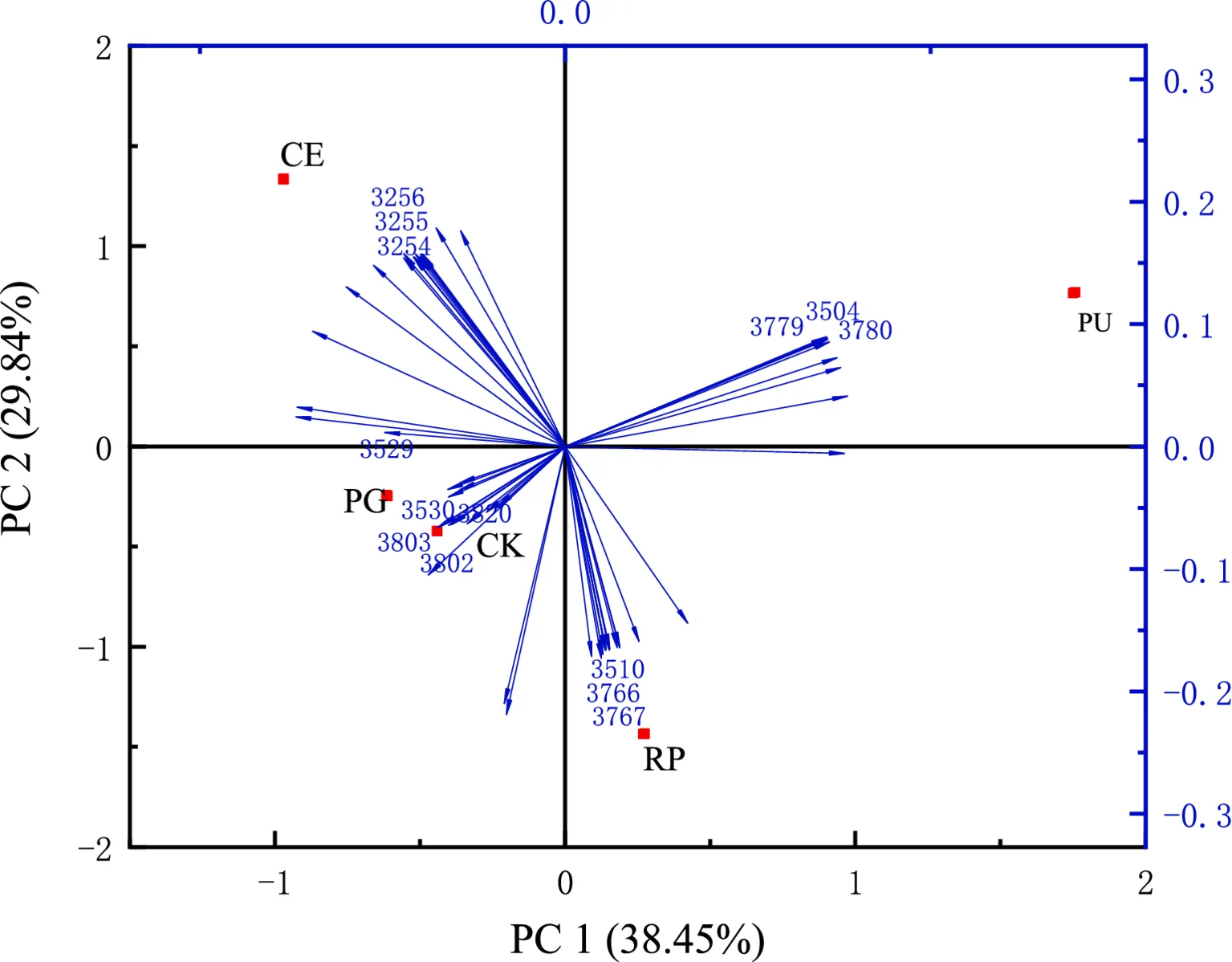
PCA biplot of electronic-eye data. CK, control bread; PU, pumpkin bread; PG, *Pueraria thomsonii* bread; RP, red pepper bread; CE, celery bread.

Loading analysis further showed clear associations between sample separation and individual color codes. PU was mainly associated with strong yellow variables. RP was primarily influenced by moderate orange yellow and moderate yellow variables, whereas CE was more closely associated with moderate yellow and grayish yellow variables. CK and PG were both related to pale yellow green and grayish yellow variables, although the contributions of these variables differed. The electronic-eye results indicate that color changes caused by the vegetable ingredients contributed substantially to the separation of the formulations in PCA space.

### 3.2 Electronic tongue analysis

The seven electronic-tongue sensors showed distinct response patterns among the vegetable breads (Figure 4I). Statistical analysis of the raw sensor responses from the three independent production batches showed significant formulation effects for AHS, PKS, CTS, NMS, CPS, ANS, and SCS (FDR-adjusted *p* < 0.01; Supplementary Table S2). CK generally showed higher responses for PKS, NMS, CPS, and ANS. RP showed higher responses for AHS, PKS, CTS, and SCS, whereas CE showed pronounced responses for CTS and SCS. PG showed relatively low responses for most sensors. These findings demonstrate clear differences in the electronic-tongue response patterns among the vegetable breads.

**Figure 4:**
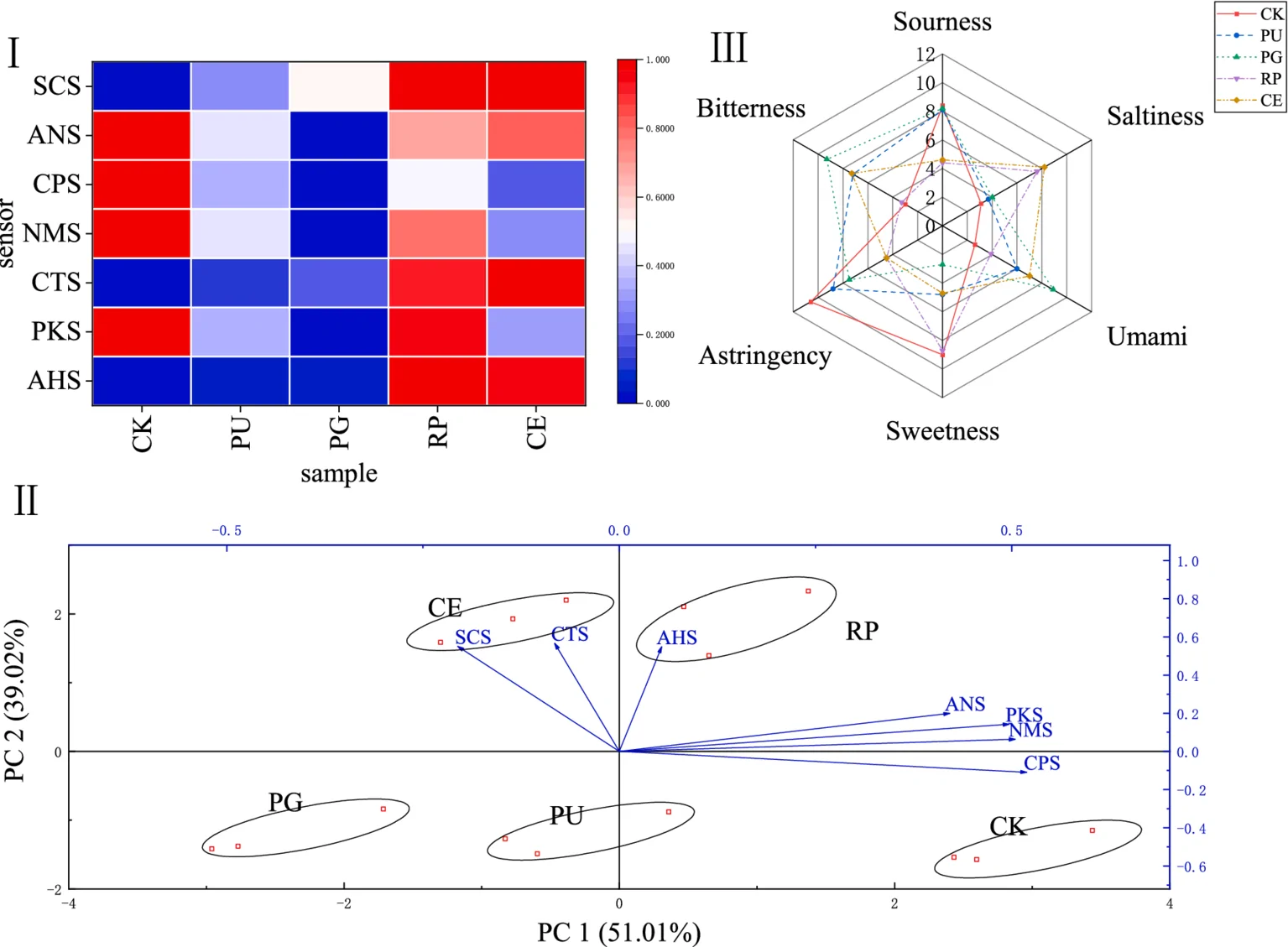
Electronic-tongue analysis of different vegetable breads. **(I)** normalized heatmap of sensor responses; **(II)** PCA biplot; **(III)** radar chart of taste-response profiles. CK, control bread; PU, pumpkin bread; PG, *Pueraria thomsonii* bread; RP, red pepper bread; CE, celery bread.

PCA was then performed on the responses of the seven sensors (Figure 4II). PC1 and PC2 explained 51.01 and 39.02% of the variance, respectively, for a cumulative explained variance of 90.03%, indicating that they captured most of the original sensor-response information. The three independent production batches within each formulation clustered well, and all five formulations occupied relatively distinct regions of the PCA space. CK, PG, RP, and CE were distributed in different quadrants, whereas PU was located below and near the origin. These results indicate that the electronic tongue effectively characterized and differentiated the overall taste-response patterns of the vegetable breads. Loading analysis showed that CK was oriented similarly to ANS, PKS, NMS, and CPS; RP was mainly associated with AHS; and CE showed clear associations with CTS and SCS. These patterns were generally consistent with the normalized heatmap. Together, the sensor responses and PCA results show that the electronic tongue can characterize differences in taste-response patterns among vegetable breads from a multisensor perspective.

Figure 4III shows the taste-response indices generated by the instrument’s Taste Ranking function. The evaluation methods and calibration scales used for sourness, saltiness, and umami differed from those used for bitterness, sweetness, and astringency. The indices were therefore used only for comparisons among formulations within the same taste attribute. PG showed relatively high umami and bitterness response indices of 8.9 and 9.3, respectively, and a relatively low sweetness response index (2.7). CE showed a relatively high saltiness response index (8.2) and also relatively high umami and bitterness responses. RP had a relatively high sweetness response index (8.7) and a low sourness response index (4.4), whereas CK showed relatively high sourness, astringency, and sweetness response indices. These values represent relative taste responses under the current extraction, sensor-response, and instrument-calibration conditions and should not be interpreted as direct measures of human-perceived taste intensity.

Differences in electronic-tongue responses among formulations may be related to water-soluble constituents introduced by the vegetable ingredients. *Pueraria thomsonii* contains abundant flavonoids, isoflavones, and other polyphenolic compounds (19), and some polyphenols have bitter sensory properties (20). These water-soluble constituents may alter the overall chemical composition of the extract and the sensor responses, potentially contributing to the higher bitterness response index of PG. However, the relevant compounds were not quantified in this study, and their specific relationships with the electronic-tongue responses require further verification. Celery contains inorganic ions and free amino acids (21, 22). Free amino acids are closely associated with umami taste (23), so these constituents may be associated with the relatively high saltiness and umami responses of CE. These explanations are literature-based hypotheses. Direct measurements of chemical composition are required to establish specific relationships between individual constituents and electronic-tongue responses.

### 3.3 Electronic nose analysis

Figure 5 shows the electronic-nose chromatographic profiles of the vegetable breads obtained on the MXT-5 and MXT-1701 columns. Multiple common chromatographic peaks were observed in all five formulations, but the number of peaks and their response intensities differed among samples in several retention-time regions. These differences indicate that the addition of pumpkin, *Pueraria thomsonii*, red pepper, and celery juices altered the overall composition and relative responses of volatile compounds in bread. Because the chromatographic profiles showed sample-dependent differences, PCA was further used to compare volatile-compound characteristics among the formulations.

**Figure 5:**
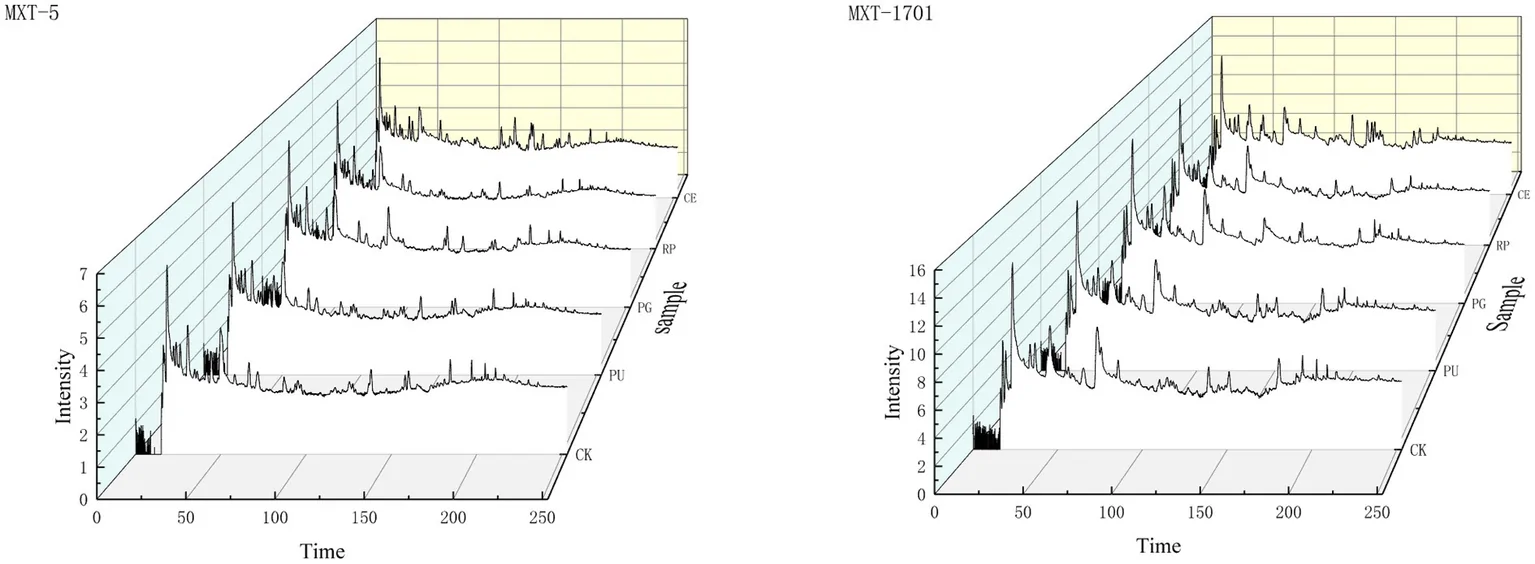
Chromatographic profiles of different vegetable breads obtained using the ultrafast gas chromatography electronic nose. CK, control bread; PU, pumpkin bread; PG, *Pueraria thomsonii* bread; RP, red pepper bread; CE, celery bread.

After ln(1 + x) transformation and autoscaling, the 97 effective electronic-nose peaks were subjected to unsupervised PCA (Figure 6I). PC1, PC2, and PC3 explained 30.14, 23.80, and 17.27% of the variance, respectively, for a cumulative explained variance of 71.20%. The first five principal components explained 82.89% of the variance and were therefore retained for subsequent feature-level data fusion. PG, CK, and CE occupied different regions of the PC1-PC2 plane, whereas PU and RP showed substantial overlap. Thus, the volatile-compound profiles of the vegetable breads showed both differences and similarities.

**Figure 6:**
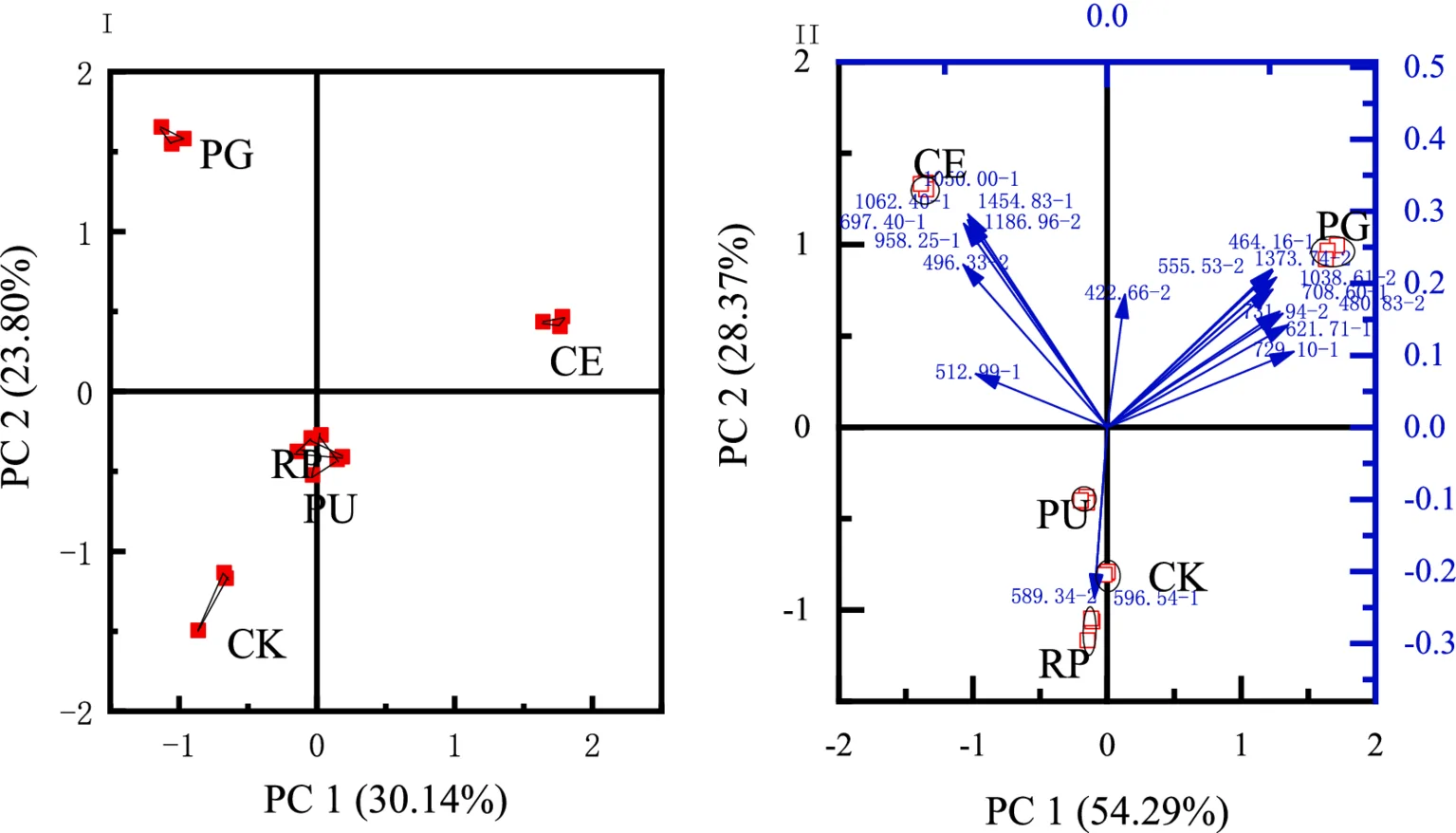
Principal component analysis of electronic-nose data from different vegetable breads. **(I)** Unsupervised PCA score plot based on all 97 effective chromatographic peaks; **(II)** exploratory PCA biplot based on the top 20 chromatographic peaks ranked by Fisher score. CK, control bread; PU, pumpkin bread; PG, *Pueraria thomsonii* bread; RP, red pepper bread; CE, celery bread. Suffixes “-1” and “-2” denote chromatographic peaks obtained from the MXT-5 and MXT-1701 columns, respectively.

The PCA biplot based on the top 20 differential chromatographic peaks ranked by Fisher score is shown in Figure 6II. PC1 and PC2 explained 54.29 and 28.37% of the variance, respectively, for a cumulative explained variance of 82.66%. The three independent batches within each formulation clustered closely, and the five formulations occupied different regions of the score plot. PG and CE were located in the first and second quadrants, respectively, and were relatively distant from the other samples, indicating more pronounced differences in their volatile profiles. Loading analysis further showed that individual volatile variables contributed differently to sample separation. The position of PG was aligned with the loading directions of ethanol (464.16–1/555.53–2), isobutanol (621.71–1/731.94–2), 3-hydroxy-2-butanone (708.60–1), isoamyl alcohol (729.10–1), and acetaldehyde (480.83–2). These differential peaks were closely aligned with the score position of PG. CE was mainly associated with pentanal (697.40–1), 1-heptanol (958.25–1), and peak 1186.96–2; the latter was tentatively matched to (−)-trans-rose oxide based on dual-column RIs. Separation of RP was mainly associated with peaks such as 589.34–2, which was tentatively assigned to acetone. Ethanol, isobutanol, and isoamyl alcohol are common volatile products of bread fermentation, whereas 3-hydroxy-2-butanone is an important bread volatile with buttery and creamy notes (24–26). Overall, the loading results indicate that the vegetable juices altered the volatile-compound composition of bread and produced distinct chromatographic response patterns. Because Fisher scores were calculated using formulation labels, this analysis was used only to interpret associations between differential variables and samples and was not considered independent evidence of discrimination performance.

Based on matches between experimental RIs on the MXT-5 and MXT-1701 columns and reference RIs in the AroChemBase database, 17 volatile compounds were tentatively assigned (Table 1). Among the top 20 chromatographic peaks ranked by Fisher score, 17 yielded candidate compound assignments that met the predefined dual-column RI criteria. The remaining three peaks had no candidate compounds that met the matching criteria and were therefore left unassigned. Because neither mass spectrometry nor authentic standards were used for structural confirmation, all compound identities were considered tentative. Peak area represented the FID response intensity and was used only for relative comparison of the same aligned chromatographic peak across formulations. Twelve of the 17 tentatively assigned compounds were recorded in all 15 independent production batches across the five formulations. Significant formulation effects on peak area were observed for all 12 compounds (FDR-adjusted *p* < 0.05).

**Table 1:** Tentative identification of volatile compounds in different vegetable breads by dual-column retention indices and distribution of peak areas.

Tentatively assigned compound	CAS	Exp. RI-5	DB RI-5	Exp. RI-1701	DB RI-1701	Cumulative ΔRI	CK peak area (a.u.)	PU peak area (a.u.)	PG peak area (a.u.)	RP peak area (a.u.)	CE peak area (a.u.)	FDR-adjusted *p*
Ethanol	64–17-5	464	448.5	555	563	23.5	642950.55 ± 78812.58 b	567517.40 ± 118539.63 b	1014221.32 ± 177707.34 a	529094.57 ± 74803.30 b	590461.84 ± 80908.12 b	0.002
Acetone	67–64-1	493	498	590	594	9	ND	ND	ND	12189.52 ± 3518.70	ND	-
1-Propanol	71–23-8	548	543	664	666.5	7.5	3050.37 ± 445.97 b	3306.50 ± 330.66 b	5388.41 ± 857.46 a	3177.39 ± 354.07 b	3407.52 ± 719.45 b	0.003
Isobutanol	78–83-1	621	626.5	733	736.5	9	11084.08 ± 1108.58 bc	8369.81 ± 789.54 cd	28391.41 ± 939.55 a	11508.72 ± 1329.20 b	7521.21 ± 1001.89 d	<0.001
3-Methylbutanal	590–86-3	648	652	726	729	7	240.67 ± 41.94 c	547.29 ± 91.12 b	562.01 ± 36.28 b	997.71 ± 188.59 a	1033.19 ± 125.33 a	<0.001
2-Methylbutanal	96–17-3	659	662	741	729	15	190.28 ± 12.74 c	480.74 ± 63.15 ab	372.68 ± 48.95 b	430.86 ± 85.12 ab	544.71 ± 66.95 a	<0.001
Pentanal	110–62-3	697	697.5	783	784	1.5	400.32 ± 66.29 c	909.35 ± 158.08 b	526.41 ± 87.98 bc	374.59 ± 46.53 c	2480.58 ± 352.88 a	<0.001
3-Hydroxy-2-butanone	513–86-0	708	712	855	856.5	5.5	1640.50 ± 185.07 c	2859.64 ± 521.11 b	5147.59 ± 271.76 a	1360.13 ± 128.51 c	1584.82 ± 198.93 c	<0.001
3-Methyl-1-butanol (isoamyl alcohol)	123–51–3	731	735.5	846	846	4.5	15971.99 ± 2510.87 b	15362.10 ± 4064.80 b	34931.62 ± 3016.62 a	18718.12 ± 1569.81 b	11680.88 ± 2302.93 b	<0.001
Hexanal	66–25-1	800	801	889	890	2	727.86 ± 70.39 c	926.36 ± 98.53 c	1440.23 ± 216.04 b	835.93 ± 108.61 c	1816.06 ± 135.61 a	<0.001
1-Hexanol	111–27-3	868	870	982	980	4	249.10 ± 5.88 b	267.01 ± 36.78 b	6557.55 ± 1074.09 a	86.66 ± 8.19 b	201.05 ± 38.41 b	<0.001
1-Heptanol	111–70-6	958	970	1,079	1,077	14	ND	95.94 ± 2.74	ND	ND	875.48 ± 151.95	-
Acetaldehyde	75–07-0	434	428.5	480	484.5	10	863.51 ± 240.15 b	1091.16 ± 184.32 b	2648.04 ± 411.07 a	1311.47 ± 151.25 b	928.51 ± 39.52 b	<0.001
Myrcene	123–35-3	991	996	1,022	1024.5	7.5	ND	ND	31.89 ± 8.44	49.50 ± 2.50	2797.32 ± 100.18	-
β-Phellandrene	555–10-2	1,033	1,031	1,063	1,059	6	402.88 ± 69.44 c	485.99 ± 43.81 c	1241.94 ± 189.22 b	330.21 ± 27.72 c	2620.50 ± 136.10 a	<0.001
(5Z)-Octa-1,5-dien-3-one	65767–22-8	980	983.5	1,085	1,085	3.5	36.49 ± 7.59	110.97 ± 19.28	16.99 ± 1.30	ND	1494.75 ± 176.26	-
(−)-trans-rose oxide	5258–11-7	1,136	1,127	1,186	1,183	12	ND	ND	ND	ND	524.53 ± 35.76	-

“-,” not applicable because the compound was not detected in all formulations. ND values were not imputed as zero, and no ANOVA or FDR adjustment was performed for compounds with incomplete data. FDR adjustment was applied only to compounds with complete peak-area data across all 15 independent production batches. Different lowercase letters within a row indicate significant differences among formulations according to Tukey’s multiple-comparison test (*p* < 0.05). CK, control bread; PU, pumpkin bread; PG, Pueraria thomsonii bread; RP, red pepper bread; CE, celery bread.

The formulations showed characteristic differences in volatile-peak responses. PG generally had higher peak responses for ethanol, isobutanol, 3-hydroxy-2-butanone, isoamyl alcohol, and acetaldehyde. Ethanol, acetaldehyde, and 3-hydroxy-2-butanone are common fermentation-related volatiles in bread (26–28), whereas higher alcohols such as isobutanol and isoamyl alcohol can be formed from branched-chain amino acids through the Ehrlich pathway in yeast (27, 29). The high responses of several fermentation-related volatiles in PG therefore suggest that its volatile profile may reflect formulation-related differences in fermentation metabolism. The underlying mechanism, however, requires further investigation, including measurements of fermentable sugars, free amino acids, and related precursors.

All three independent RP production batches showed a chromatographic peak tentatively assigned to acetone (12,189.52 ± 3,518.70 a.u.). No corresponding matched peak was recorded in the other formulations under the current analytical and database-matching conditions. Its formation may be related to thermal transformation of red-pepper-derived constituents during baking (30), but this interpretation requires further confirmation.

In CE, the peak tentatively assigned to β-phellandrene was significantly higher than in the other formulations, and the peak tentatively assigned to myrcene also showed a relatively high response in CE. Pentanal, 1-heptanol, and the peak tentatively assigned to (−)-trans-rose oxide were also prominent. Terpene-rich volatile profiles have been reported for celery (31, 32). This observation is broadly consistent with the volatile-compound characteristics observed in CE.

### 3.4 PCA based on feature-level fusion of multisource intelligent sensory data

The electronic eye, electronic tongue, and electronic nose provided information on color, taste responses, and volatile-compound profiles, respectively. The three data modalities were therefore integrated using feature-level data fusion. Based on the prespecified cumulative explained-variance threshold of ≥80%, the first three electronic-eye PCs, the first two electronic-tongue PCs, and the first five electronic-nose PCs were retained for data fusion. The PCA results for the fused dataset are shown in Figure 7. PC1, PC2, and PC3 explained 33.98, 29.08, and 17.72% of the variance, respectively, for a cumulative explained variance of 80.78%. In the PC1-PC2 plane, CK and RP were located mainly above the x-axis, whereas PG and PU were below the x-axis. CE straddled the x-axis on the negative side of PC1. The three independent production batches within each formulation showed good overall within-group consistency. These results indicate that integrating information from the three instruments within a common analytical framework preserved multidimensional differences among the vegetable breads.

**Figure 7:**
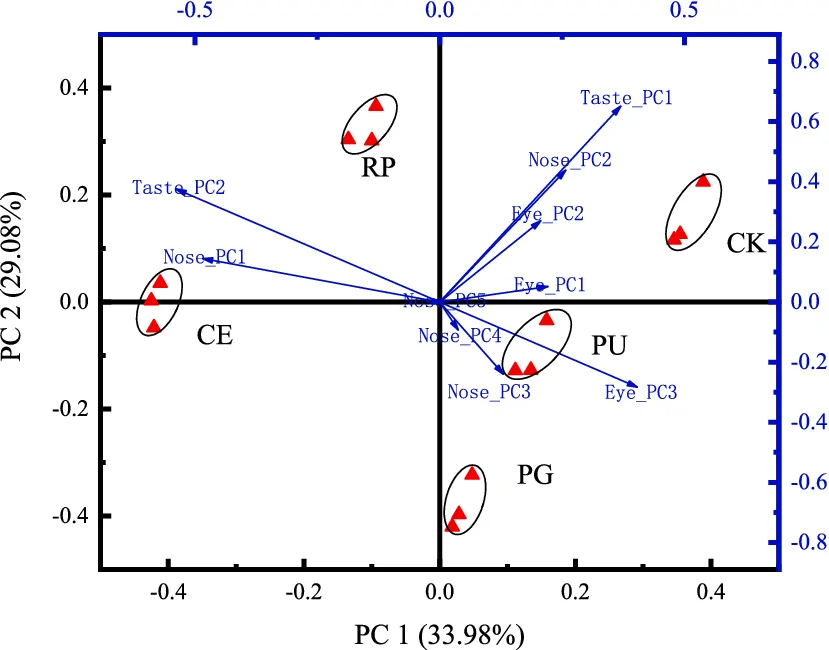
PCA biplot of feature-level fusion of multisource intelligent sensory data from different vegetable breads. CK, control bread; PU, pumpkin bread; PG, *Pueraria thomsonii* bread; RP, red pepper bread; CE, celery bread.

Loading analysis showed that fusion PC1 and PC2 were both composed of multiple latent variables from the electronic eye, electronic tongue, and electronic nose. The largest negative loadings on PC1 were Taste_PC2 (−0.538) and Nose_PC1 (−0.484). Their loading directions were close to the score positions of RP and CE, respectively; CE was more strongly aligned with Nose_PC1, whereas RP was closer to Taste_PC2. Positive PC1 loadings included Eye_PC3 (0.403), Taste_PC1 (0.370), Nose_PC2 (0.258), and Eye_PC1 (0.221). The largest positive loadings on PC2 were Taste_PC1 (0.651), Nose_PC2 (0.439), Taste_PC2 (0.376), and Eye_PC2 (0.270), which corresponded to the upper region containing CK and RP. Negative PC2 loadings were mainly associated with Eye_PC3 (−0.283), Nose_PC3 (−0.240), and Nose_PC4 (−0.093), consistent with the directions of PU and PG. Nose_PC5 had loadings close to zero on both PC1 and PC2, indicating a relatively limited contribution to the first two fusion components.

Overall, sample distributions in the fusion space were jointly characterized by latent variables related to color, taste response, and volatile-compound features. The loading directions of latent variables from the three instruments were not identical on PC1 and PC2, showing that latent variables from the three modalities contributed differently to the fused representation. Taste_PC, Nose_PC, and Eye_PC in the fusion PCA are latent variables extracted from the corresponding single-modality PCAs. Their specific meanings should therefore be interpreted in conjunction with the single-modality loading results described above.

## 4 Conclusion

This study used an electronic eye, an electronic tongue, and an ultrafast gas chromatography electronic nose to compare vegetable breads in terms of color, taste responses, and volatile-compound profiles. The three datasets were then integrated at the feature level. The electronic eye showed that PU was dominated by strong yellow and vivid yellow, RP was orange-yellow, CE was predominantly yellow but retained a green hue, and PG contained a relatively high proportion of grayish yellow. All seven electronic-tongue sensors showed significant formulation effects. Taste Ranking indicated relatively high umami and bitterness responses in PG, relatively high saltiness in CE, and relatively high sweetness in RP; however, these values represent instrumental responses under the specified analytical conditions. The electronic nose yielded 97 effective chromatographic peaks, and the first five principal components of the unsupervised PCA explained 82.89% of the variance. Dual-column RI matching yielded tentative assignments for 17 volatile compounds. PG showed high responses for several fermentation-related volatiles, RP showed a characteristic chromatographic peak tentatively assigned to acetone, and peaks tentatively assigned to myrcene and β-phellandrene showed relatively high responses in CE. In the feature-level fusion PCA, the first three principal components explained 80.78% of the variance. Loading analysis showed that multiple latent variables from all three instruments jointly contributed to the multidimensional characterization of the formulations. Overall, the vegetable juice formulations produced distinct color, taste-response, and volatile-compound characteristics in bread. Feature-level data fusion integrated the complementary information provided by the three instruments within a common data space and provided a methodological framework for the multidimensional characterization of vegetable breads.

This study has several limitations. Only three independent production batches were prepared for each formulation; therefore, the PCA and feature-level data fusion results should be viewed as exploratory, and the stability of these results requires validation using additional independent batches. Electronic-eye, electronic-tongue, and electronic-nose responses are instrumental measurements and should not be directly equated with human sensory perception. Because standardized human sensory evaluation was not included in the present study, the correspondence between the observed instrumental response patterns and human-perceived color, taste, and odor characteristics could not be directly evaluated. In addition, volatile compounds were tentatively assigned using dual-column RIs only, and FID peak areas do not provide direct measures of absolute concentration or odor contribution. The composition of the vegetable juices with respect to sugars, organic acids, inorganic ions, pigments, and other constituents was neither standardized nor measured concurrently. The observed differences therefore cannot be attributed to any single chemical constituent. Future studies should combine standardized sensory evaluation, targeted compositional analysis, and GC–MS confirmation to further validate these findings.

## Data Availability

The original contributions presented in the study are included in the article/Supplementary material, further inquiries can be directed to the corresponding authors.
