# Supplementary material for: Multidimensional characterization of vegetable breads using multisource intelligent sensory technologies and feature-level data fusion
Source: Front Nutr. 2026 Oct 13;13:1970901. doi: 10.3389/fnut.2026.1970901 (PMC13710264; doi:10.3389/fnut.2026.1970901)
Supplement: Supplementary file 1 [file Table_1.docx]

**Supplementary Material**

**Supplementary Table S1. Relative area proportions of major color codes in different vegetable bread formulations and statistical comparisons**

| **Color code** | **Color name** | **CK (%)** | **PU (%)** | **PG (%)** | **RP (%)** | **CE (%)** | **ANOVA P** | **FDR-adjusted P** |
| --- | --- | --- | --- | --- | --- | --- | --- | --- |
| 3803 | pale yellow | 32.41 ± 0.02^a^ | 0.00 ± 0.00^c^ | 17.19 ± 0.05^b^ | 0.00 ± 0.00^c^ | 0.00 ± 0.00^c^ | <0.001 | <0.001 |
| 3802 | pale yellow | 20.32 ± 0.04^a^ | 0.00 ± 0.00^c^ | 11.90 ± 0.08^b^ | 0.04 ± 0.00^c^ | 0.01 ± 0.00^c^ | <0.001 | <0.001 |
| 3820 | pale yellow green | 5.32 ± 0.02^a^ | 0.00 ± 0.00^c^ | 0.64 ± 0.01^b^ | 0.00 ± 0.00^c^ | 0.00 ± 0.00^c^ | <0.001 | <0.001 |
| 3780 | strong yellow | 0.00 ± 0.00^b^ | 10.52 ± 0.05^a^ | 0.00 ± 0.00^b^ | 0.00 ± 0.00^b^ | 0.00 ± 0.00^b^ | <0.001 | <0.001 |
| 3779 | strong yellow | 0.00 ± 0.00^b^ | 9.36 ± 0.12^a^ | 0.00 ± 0.00^b^ | 0.00 ± 0.00^b^ | 0.00 ± 0.00^b^ | <0.001 | <0.001 |
| 3504 | strong yellow | 0.00 ± 0.00^b^ | 9.17 ± 0.09^a^ | 0.00 ± 0.00^b^ | 0.00 ± 0.00^b^ | 0.00 ± 0.00^b^ | <0.001 | <0.001 |
| 3778 | strong yellow | 0.00 ± 0.00^b^ | 7.26 ± 0.13^a^ | 0.00 ± 0.00^b^ | 0.00 ± 0.00^b^ | 0.00 ± 0.00^b^ | <0.001 | <0.001 |
| 3781 | strong yellow | 0.00 ± 0.00^b^ | 7.17 ± 0.06^a^ | 0.00 ± 0.00^b^ | 0.03 ± 0.00^b^ | 0.00 ± 0.00^b^ | <0.001 | <0.001 |
| 3777 | vivid yellow | 0.00 ± 0.00^b^ | 5.68 ± 0.12^a^ | 0.00 ± 0.00^b^ | 0.00 ± 0.00^b^ | 0.00 ± 0.00^b^ | <0.001 | <0.001 |
| 3776 | vivid yellow | 0.00 ± 0.00^b^ | 5.20 ± 0.12^a^ | 0.00 ± 0.00^b^ | 0.00 ± 0.00^b^ | 0.00 ± 0.00^b^ | <0.001 | <0.001 |
| 3530 | grayish yellow | 3.11 ± 0.02^b^ | 0.00 ± 0.00^d^ | 13.82 ± 0.09^a^ | 0.03 ± 0.00^d^ | 0.58 ± 0.00^c^ | <0.001 | <0.001 |
| 3529 | grayish yellow | 3.38 ± 0.03^c^ | 0.02 ± 0.00^e^ | 11.17 ± 0.06^a^ | 0.22 ± 0.01^d^ | 4.10 ± 0.02^b^ | <0.001 | <0.001 |
| 3546 | pale greenish yellow | 0.59 ± 0.00^b^ | 0.00 ± 0.00^d^ | 5.86 ± 0.03^a^ | 0.00 ± 0.00^d^ | 0.17 ± 0.00^c^ | <0.001 | <0.001 |
| 3767 | moderate orange yellow | 0.00 ± 0.00^b^ | 0.00 ± 0.00^b^ | 0.01 ± 0.00^b^ | 11.08 ± 0.07^a^ | 0.00 ± 0.00^b^ | <0.001 | <0.001 |
| 3510 | moderate yellow | 0.12 ± 0.01^c^ | 0.57 ± 0.01^b^ | 0.14 ± 0.00^c^ | 10.84 ± 0.02^a^ | 0.12 ± 0.00^c^ | <0.001 | <0.001 |
| 3766 | moderate orange yellow | 0.00 ± 0.00^b^ | 0.01 ± 0.00^b^ | 0.00 ± 0.00^b^ | 10.00 ± 0.04^a^ | 0.00 ± 0.00^b^ | <0.001 | <0.001 |
| 3511 | moderate yellow | 0.61 ± 0.01^c^ | 0.09 ± 0.01^e^ | 0.69 ± 0.01^b^ | 9.24 ± 0.02^a^ | 0.45 ± 0.01^d^ | <0.001 | <0.001 |
| 3784 | moderate yellow | 0.35 ± 0.02^b^ | 0.03 ± 0.00^d^ | 0.20 ± 0.00^c^ | 6.47 ± 0.01^a^ | 0.00 ± 0.00^e^ | <0.001 | <0.001 |
| 3509 | strong yellow | 0.02 ± 0.00^c^ | 1.72 ± 0.00^b^ | 0.03 ± 0.00^c^ | 5.71 ± 0.04^a^ | 0.01 ± 0.00^c^ | <0.001 | <0.001 |
| 3255 | moderate yellow | 0.41 ± 0.00^c^ | 0.12 ± 0.00^d^ | 1.00 ± 0.02^b^ | 0.06 ± 0.00^d^ | 20.05 ± 0.06^a^ | <0.001 | <0.001 |
| 3256 | grayish yellow | 0.54 ± 0.00^c^ | 0.05 ± 0.00^d^ | 1.26 ± 0.01^b^ | 0.06 ± 0.00^d^ | 12.06 ± 0.03^a^ | <0.001 | <0.001 |
| 3254 | moderate yellow | 0.07 ± 0.00^c^ | 0.22 ± 0.01^b^ | 0.22 ± 0.01^b^ | 0.02 ± 0.00^d^ | 8.96 ± 0.01^a^ | <0.001 | <0.001 |
| 3272 | grayish greenish yellow | 0.00 ± 0.00^d^ | 0.02 ± 0.00^c^ | 0.06 ± 0.00^b^ | 0.00 ± 0.00^d^ | 7.66 ± 0.01^a^ | <0.001 | <0.001 |
| 3528 | moderate yellow | 1.07 ± 0.00^c^ | 0.13 ± 0.01^e^ | 2.81 ± 0.05^b^ | 0.37 ± 0.02^d^ | 5.09 ± 0.01^a^ | <0.001 | <0.001 |

**Note:** Values are presented as mean ± SD (n = 3 independent production batches). Relative area proportions are expressed as percentages. Different lowercase superscript letters within the same row indicate significant differences among formulations according to Tukey's HSD test (P < 0.05). ANOVA P values were adjusted across the 24 color-code variables using the Benjamini-Hochberg procedure to control the false discovery rate (FDR). P values < 0.001 are reported as <0.001. CK, control bread; PU, pumpkin bread; PG, Pueraria thomsonii bread; RP, red pepper bread; CE, celery bread.

**Supplementary Table S2. Electronic-tongue sensor responses in different vegetable bread formulations and statistical comparisons**

| **Sensor** | **CK** | **PU** | **PG** | **RP** | **CE** | **F(4,10)** | **ANOVA P** | **FDR-adjusted P** | **η²** | **Interpretation** |
| --- | --- | --- | --- | --- | --- | --- | --- | --- | --- | --- |
| AHS | 3528.01 ± 23.29^c^ | 3533.66 ± 26.37^bc^ | 3532.24 ± 26.67^bc^ | 3615.40 ± 33.90^a^ | 3612.38 ± 38.60^ab^ | 6.707 | 0.007 | 0.007 | 0.728 | Significant formulation effect |
| PKS | 1800.93 ± 6.20^a^ | 1765.67 ± 8.35^b^ | 1747.94 ± 9.23^b^ | 1798.67 ± 5.51^a^ | 1764.74 ± 3.91^b^ | 33.819 | <0.001 | <0.001 | 0.931 | Significant formulation effect |
| CTS | 874.45 ± 3.45^b^ | 888.76 ± 13.99^b^ | 893.96 ± 5.33^b^ | 976.18 ± 42.61^a^ | 987.37 ± 12.67^a^ | 19.220 | <0.001 | <0.001 | 0.885 | Significant formulation effect |
| NMS | 1583.11 ± 16.54^a^ | 1543.58 ± 18.83^abc^ | 1509.53 ± 19.10^c^ | 1568.38 ± 14.81^ab^ | 1531.33 ± 16.54^bc^ | 8.637 | 0.003 | 0.003 | 0.776 | Significant formulation effect |
| CPS | 2318.61 ± 10.96^a^ | 2224.27 ± 11.62^bc^ | 2171.95 ± 12.82^d^ | 2241.40 ± 10.00^b^ | 2200.38 ± 10.23^cd^ | 73.618 | <0.001 | <0.001 | 0.967 | Significant formulation effect |
| ANS | 1482.79 ± 5.50^a^ | 1431.61 ± 4.95^c^ | 1388.67 ± 4.42^d^ | 1453.61 ± 4.80^b^ | 1465.44 ± 5.74^b^ | 151.717 | <0.001 | <0.001 | 0.984 | Significant formulation effect |
| SCS | 3977.86 ± 27.56^d^ | 4109.72 ± 15.83^c^ | 4204.90 ± 5.36^b^ | 4420.25 ± 2.59^a^ | 4424.15 ± 5.14^a^ | 533.559 | <0.001 | <0.001 | 0.995 | Significant formulation effect |

**Note:** Values are presented as mean ± SD (n = 3 independent production batches). Different lowercase superscript letters within the same row indicate significant differences among formulations according to Tukey's HSD test (P < 0.05). F(4,10) is the one-way ANOVA F statistic, and η² denotes eta squared. ANOVA P values were adjusted across the seven sensor variables using the Benjamini-Hochberg procedure to control the false discovery rate (FDR). P values < 0.001 are reported as <0.001. CK, control bread; PU, pumpkin bread; PG, Pueraria thomsonii bread; RP, red pepper bread; CE, celery bread.
